# Lysine-specific demethylase 1 controls key OSCC preneoplasia inducer STAT3 through CDK7 phosphorylation during oncogenic progression and immunosuppression

**DOI:** 10.1038/s41368-025-00363-x

**Published:** 2025-04-17

**Authors:** Amit Kumar Chakraborty, Rajnikant Dilip Raut, Kisa Iqbal, Chumki Choudhury, Thabet Alhousami, Sami Chogle, Alexa S. Acosta, Lana Fagman, Kelly Deabold, Marilia Takada, Bikash Sahay, Vikas Kumar, Manish V. Bais

**Affiliations:** 1 Department of Translational Dental Medicine, Boston University Henry M. Goldman School of Dental Medicine, Boston, USA; 2 Department of Endodontics, Henry M. Goldman School of Dental Medicine, Boston University, Boston, USA; 3 College of Veterinary Medicine, University of Florida, Gainesville, USA; 4 Dept. of Biochemistry and Molecular Biotechnology, University of Massachusetts Chan Medical School, Shrewsbury, USA; 5 Present Address: Department of Basic and Clinical Oral Sciences, Faculty of Dentistry, Umm Al-Qura University, Makkah, Saudi Arabia

**Keywords:** Cancer prevention, Oral cancer

## Abstract

Oral squamous cell carcinoma (OSCC) progresses from preneoplastic precursors via genetic and epigenetic alterations. Previous studies have focused on the treatment of terminally developed OSCC. However, the role of epigenetic regulators as therapeutic targets during the transition from preneoplastic precursors to OSCC has not been well studied. Our study identified lysine-specific demethylase 1 (LSD1) as a crucial promoter of OSCC, demonstrating that its knockout or pharmacological inhibition in mice reversed OSCC preneoplasia. LSD1 inhibition by SP2509 disrupted cell cycle, reduced immunosuppression, and enhanced CD4+ and CD8+ T-cell infiltration. In a feline model of spontaneous OSCC, a clinical LSD1 inhibitor (Seclidemstat or SP2577) was found to be safe and effectively inhibit the STAT3 network. Mechanistic studies revealed that LSD1 drives OSCC progression through STAT3 signaling, which is regulated by phosphorylation of the cell cycle mediator CDK7 and immunosuppressive CTLA4. Notably, LSD1 inhibition reduced the phosphorylation of CDK7 at Tyr170 and eIF4B at Ser422, offering insights into a novel mechanism by which LSD1 regulates the preneoplastic-to-OSCC transition. This study provides a deeper understanding of OSCC progression and highlights LSD1 as a potential therapeutic target for controlling OSCC progression from preneoplastic lesions.

## Introduction

Oral squamous cell carcinoma (OSCC) is an aggressive type of cancer. Tongue dysplasia and preneoplasia originate from the epithelial layer and migrate to the adjacent tissues. Dysplastic epithelial changes in the tongue are confined to the overlying epithelium of OSCC and are characterized by infiltration of the underlying connective tissue.^1^ Recent statistics show that oral cavity-related cancers account for 53 000 cases and 10 860 deaths annually in the United States.^2^ The limited understanding of OSCC preneoplasia, as well as its mechanisms and progression, interferes with early detection and intervention.

Aberrant epigenetic reprogramming and clonal proliferation of cancer stem cells promote cancer.^3^ Precancerous lesions have diverse signaling mechanisms and immune infiltration, and there is limited understanding of which lesions progress to invasive lesions.^4–6^ A recent study implied predictability in the earliest stages of tumorigenesis and showed evolutionary constraints and barriers to malignant transformation, with implications for earlier detection and interception of aggressive, genome-unstable tumors.^7^ OSCC often develops from precancerous lesions through a multistep process that involves genetic and epigenetic changes. Previous studies have shown that OSCC often develops from precancerous lesions through a multi-step process that includes EGFR overexpression,^8^ histone modifications.^9^ and loss of cell cycle regulation.^10^ However, all of these mechanisms are observed in OSCC and not in precancer, which could be different. Investigating small molecules and drugs that target epigenetic changes could open new avenues for treatment and prevention, especially for early-stage diseases.

A proteogenomic study on clinical cancer demonstrated that lysine-specific demethylase 1 (LSD1), encoded by *KDM1A* gene, is critical in Lung Squamous Cell Carcinoma (LSCC) and head and neck cancer and shares tissue and cell type of origin.^11^ LSD1 controls SOX2 expression and is currently being investigated in a clinical context in conjunction with immunotherapy to reduce SOX2 expression in LSCC (NCT04350463). Our study showed that the aberrantly upregulated epigenetic regulator, LSD1, promotes OSCC development. LSD1 expression increases during dysplasia and progressively increases with advanced tumor grade and stage in mouse and human OSCC.^12,13^ LSD1 promotes cancer stem cells,^14^ chemoresistance, and relapse.^15^ LSD1 attenuation inhibits patient-derived xenografts and epidermal growth factor receptor (EGFR) and yes-associated protein (YAP) signaling, which are critical in OSCC.^12,16^ However, how LSD1-induced epigenetic changes reprogram preneoplasia into OSCC by acting on specific gene networks, phosphoprotein activation, and immune cells remains unclear.

Constitutive activation of signal transducer and activator of transcription 3 (STAT3) in OSCC preneoplasia is predicted to initiate malignant transformation.^17–19^ STAT3 is a transcription factor that is involved in this process. Various oncogenic pathways converge to STAT3. Although STAT3 is an attractive target, it is difficult to target OSCC cells because of its various modes of regulation.^20,21^ The interaction between EGFR and STAT3 promotes malignancy.^22^ This study aimed to evaluate how LSD1 initiates preneoplastic changes by activating early events such as STAT3 signaling, cell cycle mediators, and tumor immunity, which are useful for understanding progressive OSCC lesions from precancer and therapeutic intervention. STAT3 signaling regulates antitumor immunity and promotes an immunosuppressive tumor environment. CTLA4 induces immunosuppression and is a target for various anticancer therapies.^23–25^

Studies have shown that tobacco carcinogen, 4 nitro-quinolone-1-oxide (4NQO)-induced mouse model,^26^ and spontaneously occurring feline OSCC are similar to human OSCC. The feline OSCC model closely recapitulates several important cases of human papillomavirus-negative (HPV-ve) OSCC.^27–29^ The LSD1 inhibitor SP2509 is similar to its clinical candidate SP2577 (Seclidemstat; Salarius Pharmaceutical). Its safety and efficacy have been evaluated in phase 1/2 clinical trials,^30^ and it can be used in translational studies. Feline OSCC shares common aspects of molecular and cellular pathology, including the expression of tyrosine kinase receptors, neo-angiogenesis, inflammatory pathways, and immune cell markers.^27–29^

Here, we evaluated the following specific mechanisms in OSCC preneoplasia: 1) LSD1 regulates CDK7 and STAT3 to facilitate cell cycle progression; 2) the LSD1 promotes phosphorylation of specific cyclin-dependent kinases (CDKs) and eukaryotic translation initiation factors (eIFs); and 3) LSD1 inhibition attenuates cytotoxic T lymphocyte-associated protein 4 (CTLA4) and promotes CD8+ T cell-mediated accumulation and activation through IFNγ production. Finally, proof-of-concept studies in feline spontaneous OSCC were conducted to determine the safety of Seclidemstat and its efficacy in attenuating STAT3 mechanism in veterinary trials. Our studies in murine and feline OSCC models, transcriptional and phospho-proteomic analyses, and their correlation with human OSCC demonstrated that LSD1 plays a key role in attenuating the novel CDK7-STAT3-CTLA4 axis and promoting CD8+ T cell infiltration in OSCC preneoplasia.

## Results

### LSD1 inhibitor (SP2509) reverses cancer cell division and promotes immune response in OSCC preneoplasia

**Fig. 1:**
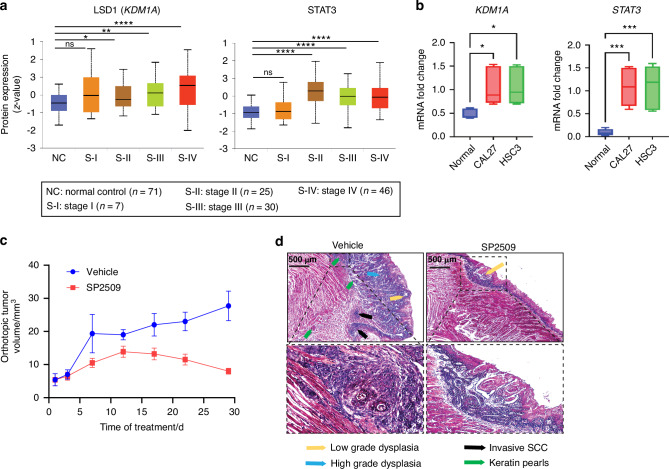
STAT3 inhibition by SP2509 attenuates orthotopic tongue tumors: 4MOSC1 primary cells derived from tobacco carcinogen 4NQO-subjected tongue tumors were injected orthotopically and treated with SP2509. RNA-seq analysis after treatment of SP2509 in orthotopic 4MOSC1 syngeneic model. **a** Clinical cancer stage protein expression of LSD1 and STAT3 from CPTAC database. **b** Fold change of *KDM1A* and *STAT3* in cancer cells like CAL27 and HSC3 in comparison with normal epithelial cells. **c** The tongue tumor volume of mice topically treated with SP2509 inhibits tumor volume. **d** The gross pathological phenotype, H&E staining of tongue treated with SP2509, showing reduced pathological lesions. “ns” *P* > 0.05, * *P* < 0.05, ** *P* < 0.01, *** *P* < 0.001, **** *P* < 0.000 1

**Fig. 2:**
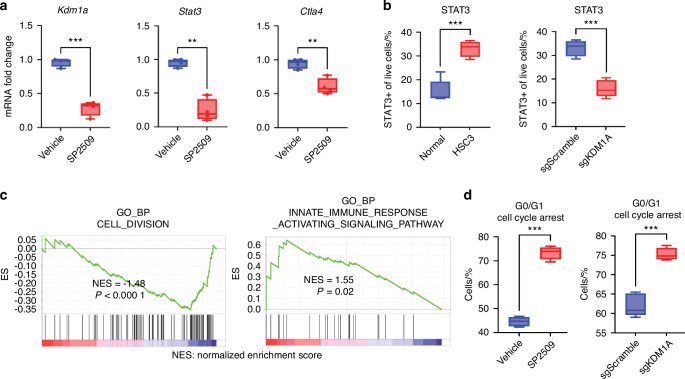
STAT3 inhibition by SP2509 reduced cell cycle progression and promotes immune response. **a** Fold change of *Kdm1a, Stat3*, and *Ctla4* mRNA expression after treatment with SP2509 in mice OSCC preneoplasia. **b** Altered STAT3 protein expression in HSC3 compared to normal epithelial cells, and *KDM1A* knockout. **c** Gene set enrichment analysis (gene ontology) shows reduction in cell division and elevation in immune response when treated with SP2509. **d** Validation of G0/G1 cell cycle arrest after SP2509 and sg*KDM1A* treatment to HSC3 cells. Statistical analyses were performed using a t-test and one-way ANOVA. “ns” *P* > 0.05, *P < 0.05, **P < 0.01, ***P < 0.001, ****P < 0.000 1

The analysis of STAT3 and LSD1 protein expression in OSCC patient samples from clinical proteomic tumor analysis consortium (CPTAC) identified that LSD1 and STAT3 protein expression increased with the progressive clinical stages (Fig. 1a) and pathological grades (Fig. S1a, b) in OSCC. RT-qPCR analysis confirmed that *KDM1A* and *STAT3* expression levels were substantially higher in cancer cells (HSC3 and CAL27) than in normal epithelial cells (Fig. 1b). These two cell lines have been extensively characterized in vivo and in vitro.^13,31^ Hub gene detection analysis of differentially expressed genes from the Cancer Genome Atlas (TCGA) patient data, which identified the relationship between the top 10 hub genes, showed that KDM1A has a primary interaction with STAT3, SOX2, EZH2, and HIF1A and a secondary interaction with EGFR (Fig. S1c, d; Tables S1 and S2 for differentially expressed genes and patient characteristics). To determine whether LSD1 inhibition reversed OSCC and STAT3, SP2509 was tested in a syngeneic OSCC mouse model. Treatment with SP2509 significantly inhibited tumor growth (Fig. 1c), and H&E staining revealed a reduction in tumor pathological changes (Fig. 1d). SP2509 also regulated *Kdm1a*, *Stat3*, and *Ctla4* expression (Fig. 2a). STAT3 protein levels were significantly higher in cancer cells than in normal epithelial cells and were significantly reduced in cancer cells when *KDM1A* was knocked out using the CRISPR-Cas9 system (Fig. 2b). RNA-Seq analysis revealed that SP2509 treatment altered the expression of a subset of genes (Fig. S1f, Table S3). Gene Ontology (GO) analysis using the Gene Set Enrichment Analysis (GSEA) tool showed negative enrichment of cell division and cell cycle process gene network, whereas positive enrichment of humoral and innate immune response network with SP2509 treatment compared with the vehicle control (Figs. 2c, S1g). To validate the effect of LSD1 inhibition and *KDM1A* knockout on the cell cycle process in vitro, we performed a Propidium Iodide (PI) staining assay using the HSC3 cell line and found that both LSD1 inhibition and *KDM1A* knockout induced G0/G1-phase cell cycle arrest, thus confirming our in vivo findings (Figs. 2d; S1h, i). Additionally, OSCC preneoplasia data^32^ reanalysis showed that *KDM1A*, *STAT3*, and *CTLA4* expression progressively increased in preneoplasia tissues compared to that in normal tissues (Fig. S2a; Table S4). GSEA Hallmark analysis showed that the *STAT3* network was upregulated in human dysplasia samples compared with that in normal samples (Fig. S2b). Ingenuity pathway analysis (IPA) revealed increased *STAT3* and an immunosuppressive network and increased G1 to S-phase cell cycle progression (Fig. S2c, d), and interestingly, this correlates with our in vitro cell cycle analysis validation. Moreover, Tumor, Normal, and Metastasis (TNM) plot analysis from TCGA showed that the expression of *KDM1A* network genes, including *STAT3* and *CTLA4*, was higher in HNSCC clinical tumors than in normal human tissues (Fig. S2e).

### LSD1 reprograms tumor microenvironment to immunosuppression

**Fig. 3:**
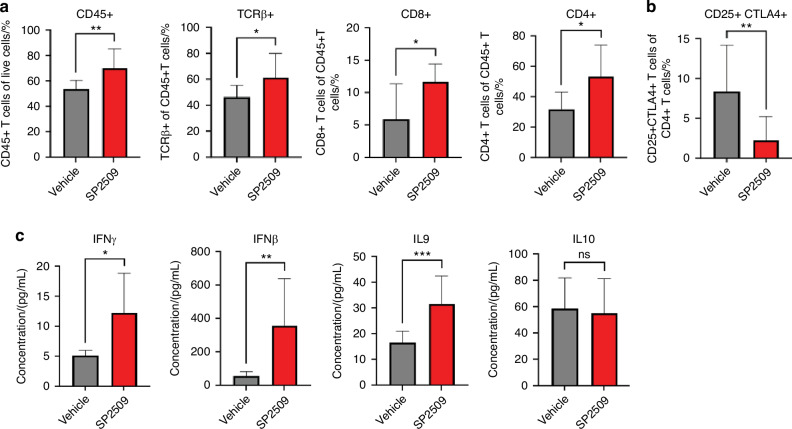
SP2509 has a unique mechanism to promote infiltration of CD8+ T cells in tongue OSCC and inhibiting immunosuppressive CTLA4+ CD25+ T cells: LSD1 regulates tumor immune microenvironment and inhibition with SP2509 facilitates infiltration of various immune cell types: **a** SP2509 promotes infiltration of CD45+, TCRβ+, CD4+, and CD8+ T cells, and **b** Reduces CTLA4+ CD25+ (of CD4+ T cells) immunosuppressive cells in OSCC. **c** Secreted cytokines measured from serum samples showing a significant increase in proinflammatory cytokines like IFNβ, IFNγ, and IL9 in the SP2509 treatment group. “ns” *P* > 0.05, *P < 0.05, **P < 0.01, ***P < 0.001, ****P < 0.000 1

**Fig. 4:**
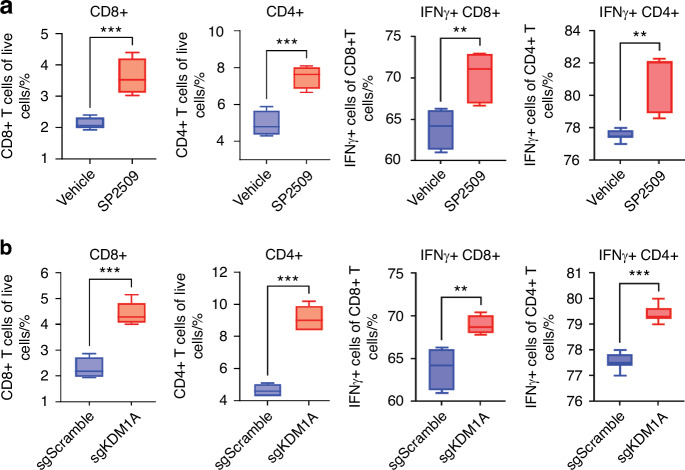
SP2509 treatment increases IFNγ producing T cells in a co-culture model. **a** Increased levels of CD8+ and CD4+ T cells along with IFNγ (in CD8+ and CD4+ T cells) in SP2509 treated HSC3 and human PBMC co-culture model. **b** Increased levels of CD8+ and CD4+ T cells along with IFNγ (in CD8+ and CD4+ T cells) in *KDM1A* deleted HSC3 and human PBMC co-culture model. “ns” *P* > 0.05, * *P* < 0.05, ** *P* < 0.01, *** *P* < 0.001, **** *P* < 0.000 1

To evaluate immune response upon LSD1 inhibition, flow cytometry analysis of single-cell suspensions from tongue tumors and spleens was performed to identify gate-specific infiltrating cells (Fig. S2f). The data showed that SP2509 treatment promoted the infiltration of CD45+, TCRβ+, CD4+, and CD8+ T cells in tongue tumors (Fig. 3a). A previous study found that CD4 + T cells regulate other immune cells, such as CD8+ T cells, where activated CD8+ T cell subsets produce various cytokines that affect the tumor microenvironment.^33^ Interestingly, SP2509 attenuated the immunosuppressive CD25+ CTLA4+ T cells (a subset of CD4+ T cells) (Fig. 3b). To evaluate the systemic effects, multiplex cytokine analysis of serum and flow cytometry of spleen immune cells from 4MOSC1 mice treated with SP2509 was performed. Interferon γ (IFN-γ) promotes antitumor immunity.^34^ Serum cytokine analysis showed that SP2509 treated mouse serum upregulated IFNβ, IFNγ, and IL9, which are known to promote proliferation and activation of CD8 + T cells (Fig. 3c). Spleen cell analysis showed the upregulation of CD45+, TCRβ+, CD4+, and CD8 + T cells (Fig. S3a). To evaluate IFNγ production status, HSC3 co-culture with human PBMCs treated with LSD1 inhibitor (Fig. 4a) and *KDM1A* knockout (Fig. 4b) in HSC3 cells showed a significant increase in IFNγ+ CD4+ and IFNγ+ CD8+ T cells compared to the respective controls.

**Fig. 5:**
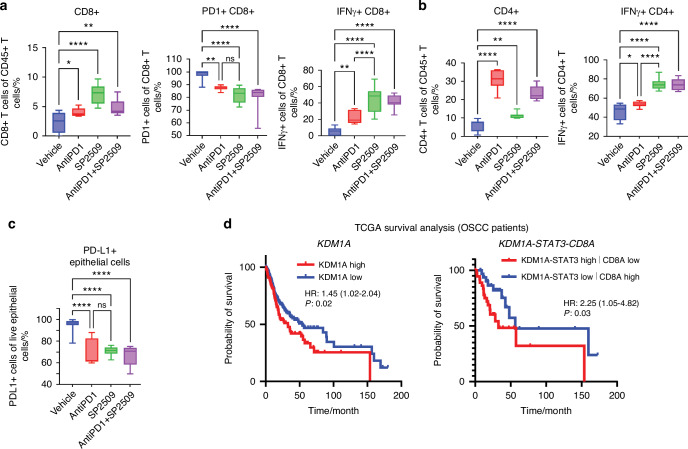
In combination with antiPD1 immunotherapy, LSD1 inhibition shows significant CD8+ T cell infiltration and activation: (a-b) 4NQO mice model treated with AntiPD1, SP2509, and AntiPD1 + SP2509 showing; **a** Variable levels of CD8+ T cells, PD1+ CD8+ T cells, and IFNγ+ CD8+ T cells, **b** Variable levels of CD4+ T cells, and IFNγ+ CD4+ T cells, and **c** Reduced levels of PD-L1+ epithelial cells. **d** Kaplan-Meier survival analysis showing reduced overall survival in *KDM1A* high, and *KDM1A-STAT3* high|*CD8A* low expressing patient groups. Statistical analyses were performed by t-test, one-way ANOVA, and Log-rank t-test. “ns” *P* > 0.05, * *P* < 0.05, ** *P* < 0.01, *** *P* < 0.001, **** *P* < 0.000 1

To assess the effect of anti-PD1 treatment (InVivoMAb; BioXCell #BE0273) in combination with SP2509, we performed flow cytometry on 4NQO-induced oral cancer mice. We observed that both CD4+ and CD8+ T cells were significantly increased in the treatment groups, and IFNγ+ CD4+ and IFNγ+ CD8+ T cells were significantly increased. Conversely, the PD1+ CD8+ T cell population decreased significantly in both treatment groups, suggesting that LSD1 inhibition has a direct role in PD1 regulation (Fig. 5a, b). It was also observed that PD-L1+ epithelial cells were also significantly decreased in all the treatment groups (Fig. 5c). Moreover, analysis of 15-year survival data from TCGA (only patients with OSCC) showed that the clinical survival of patients with *KDM1A* and *STAT3* expression and *CD8A* low expression was poor, similar to that of untreated tumors. Survival analysis resembling the SP2509 effect as *KDM1A* low, *STAT3* low, and *CD8A* high significantly increased overall survival (Fig. 5d). Additionally, patients with *KDM1A* high expression had lower survival than *KDM1A* low expression (Fig. 5d). However, *KDM1A* (high expression), *STAT3* (high expression), *KDM1A* (high expression), and *CD8A* (low expression) showed a slightly significant effect on overall survival (Fig. S3b). These findings strongly support our hypothesis that LSD1 promoted network can predict patient survival; however, this must be tested in clinical studies.

### LSD1 inhibition reverses feline spontaneous clinical OSCC by attenuating STAT3 in veterinary clinical trials

**Fig. 6:**
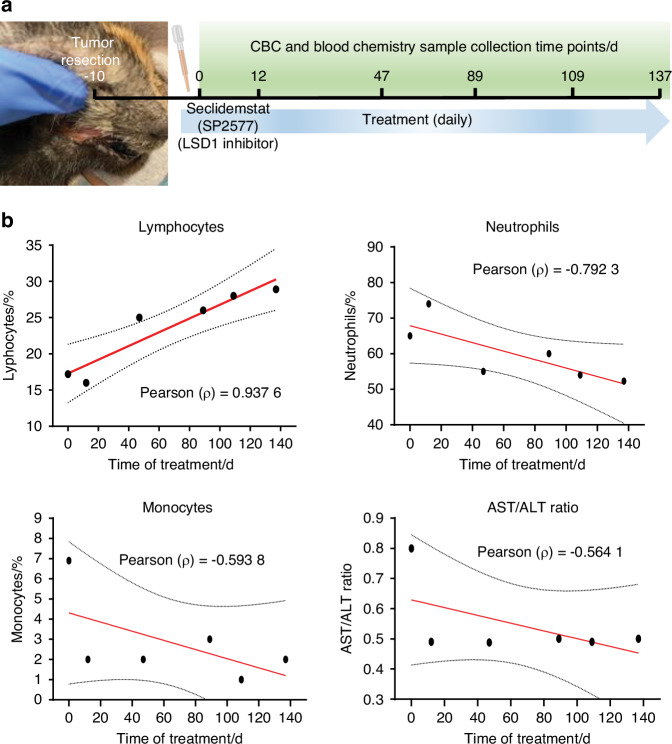
Proof of principle study showing feline spontaneous natural OSCC post-treatment with Seclidemstat shows safety of LSD1 inhibition compared to pre-treatment: To evaluate safety and mechanism of LSD1, Seclidemstat applied to client-owned cats in veterinary clinical studies. **a** Illustration of experimental design to evaluate the safety of Seclidemstat in an 11-year-old female owner-owned cat that had surgically excised primary OSCC, treated with Seclidemstat post-10 days of surgery, and analyzed for veterinary clinical parameters. **b** Complete blood count (CBC) and blood chemistry panel show increased lymphocytes, reduced monocytes and neutrophils, and reduced AST/ALT ratio (Pearson correlation)

To evaluate the efficacy of LSD1 inhibition in clinical settings, the first feline preclinical study was performed on two feline OSCC patients. This study evaluated the safety and efficacy of another LSD1 inhibitor, seclidemstat (SP2577). We recruited an 11-year-old female owner-owned cat who presented with primary OSCC that was surgically excised. To evaluate safety, the cat was treated with 10 mg/kg of Seclidemstat 10 d after surgery. The samples were analyzed for veterinary clinical parameters such as complete blood count (CBC) and blood chemistry panel during the routine visit (Fig. 6a). The data showed that Seclidemstat promoted a gradual increase in lymphocytes to the normal range but reduced monocytes and neutrophils. Additionally, the AST/ALT ratio, which could be a predictor of cancer,^35–38^ was reduced (Fig. 6b). Visible relapsed or refractory OSCC was not detected even after six months of observation.

**Fig. 7:**
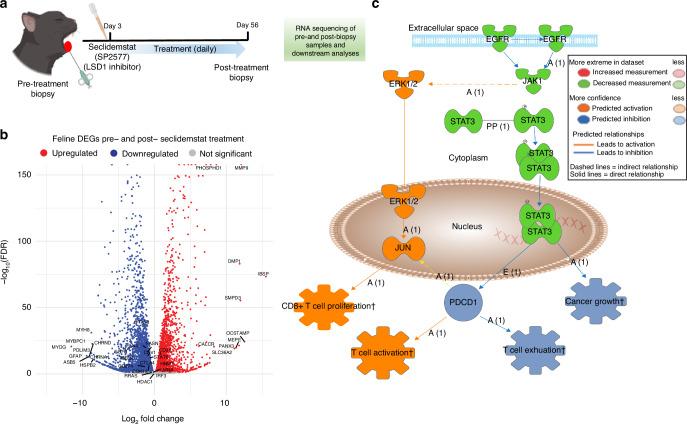
Feline spontaneous natural OSCC post-treatment with Seclidemstat shows efficacy of LSD1 inhibition compared to pre-treatment: **a** Illustration of an experimental plan to evaluate the Seclidemstat-inhibited STAT3 network in feline patients with progressive OSCC and visible tongue tumors recruited to study and treated with Seclidemstat for 56 days. **b** Volcano plot representing differentially expressed genes in pre- and post-Seclidemstat treatment highlighted significant downregulation of *STAT3, EGFR*, and immune checkpoint gene *CTLA4*, whereas *IRF3* was upregulated. **c** IPA analysis showed that downregulation in the EGFR-STAT3 pathway leads to CD8+ T cell proliferation and activation. “ns” *P* > 0.05, * *P* < 0.05, ** *P* < 0.01, *** *P* < 0.001, **** *P* < 0.000 1

To evaluate whether Seclidemstat reverses the STAT3 network, a new feline patient with progressive OSCC and visible tongue tumors was recruited and treated for 56 days (Fig. 7a). The biopsy samples were collected before and after treatment and subjected to RNA-seq, which showed that Seclidemstat attenuated the STAT3 network and was one of the top 10 hub genes (Fig. S3d, e; Table S5). Moreover, Seclidemstat attenuated *STAT3*, *CTLA4*, and *EGFR* expression, but increased *IRF3* expression (Fig. 7b). IPA revealed that seclidemstat attenuated the *EGFR-STAT3* network, reduced cancer cell growth and T cell exhaustion, and upregulated T cell activation and proliferation (Fig. 7c). Overall, seclidemstat-mediated LSD1 inhibition affected OSCC progression in cats.

### *KDM1A* knockout or LSD1 pharmacological inhibition in mice tongue attenuates OSCC preneoplasia and STAT3 phosphorylation

**Fig. 8:**
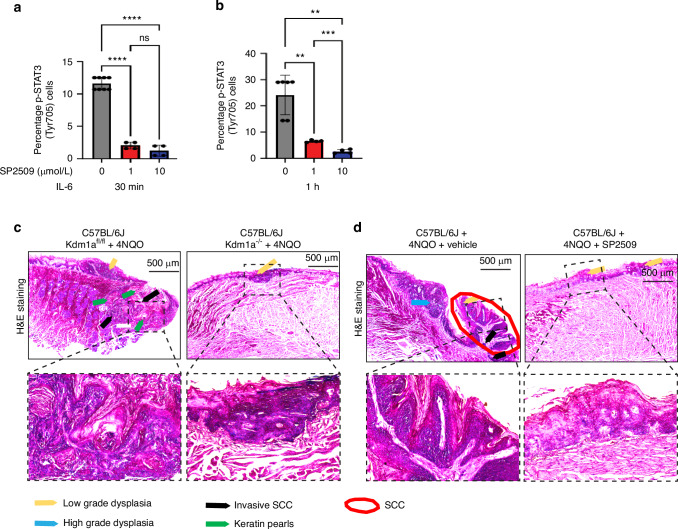
LSD1 promotes STAT3 mediated OSCC progression: **a**, **b** HSC3 cells treated with SP2509 for 24 h and IL6 for 30- and 60 min shows reduced phosphorylated STAT3 (Tyr705) levels as evaluated by phospho-flow cytometry. **c** H&E staining of tongue tissue sections in *Kdm1a*^*−/−*^+4NQO shows a reduction in the pathological lesion on the tongue after 18 weeks compared to the *Kdm1a*^fl/fl^ + 4NQO control. **d** H&E staining of tongue tissue sections isolated from mice with topical application of SP2509 inhibits pathological features of OSCC (quantification and evaluation performed blindly by a board-certified oral pathologist). Statistical analyses were performed by t-test and one-way ANOVA. “ns” *P* > 0.05, * *P* < 0.05, ** *P* < 0.01, *** *P* < 0.001, **** *P* < 0.000 1

**Fig. 9:**
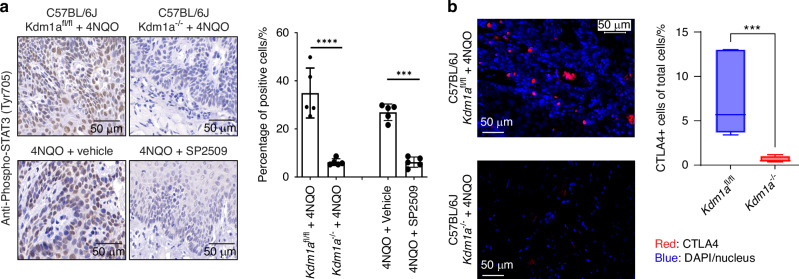
LSD1 promotes STAT3 phosphorylation and CTLA4+ immune cells: **a** Phosho-STAT3 (Tyr705) immunostaining of *Kdm1a*^*−/−*^ + 4NQO and *Kdm1a*^fl/fl^ + 4NQO (upper panel), and *Kdm1a*^fl/fl^ + 4NQO + SP2509 and *Kdm1a*^fl/fl^ + 4NQO+Vehicle treated C57BL/6J mice (lower panel), and their respective quantifications. **b** Accumulation and quantification of CTLA4+ immune cells at the tumor site in *Kdm1a*^fl/fl^ and *Kdm1a*^*−/−*^ mice tongue. Statistical analyses were performed by t-test. “ns” *P* > 0.05, *P < 0.05, ** *P* < 0.01, *** *P* < 0.001, **** *P* < 0.000 1

To evaluate whether there was a correlation between STAT3 phosphorylation and LSD1 activity in human tumors, phospho-STAT3 levels were measured by phospho-flow cytometry. Overnight exposure of SP2509 to HSC3 and CAL27 (OSCC cell lines) treated with the STAT3 activator IL6 at 30 min and 1 h had a negative impact on the phosphorylation of STAT3, where it was observed that SP2509 treatment significantly reduced STAT3 phosphorylation at Tyr705 (Fig. 8a, b) (please also see Fig. S4a for CAL27 treatment with IL6 for 1 h). Experimental design showing Keratin promoter 14 specific conditional *Kdm1a* knockout in mice tongue epithelium to evaluate the effect of genetic deletions LSD1, as well as pharmacological inhibition using SP2509 in the 4NQO mouse model were evaluated in OSCC precancer (Fig. S4b). *Kdm1a*^*−/−*^ mouse tongue tissues showed reduced OSCC pathology at week 18 post-4NQO treatment compared to *Kdm1a*^*fl/fl*^ mice (Figs. 8c; S4c). Similarly, SP2509 application during dysplasia prevented the progression of OSCC preneoplasia (Figs. 8d; S4c) as well as OSCC pathological lesions and reduced high-grade dysplasia and squamous cell carcinoma in the group treated with SP2509. Quantification of percentages in the cohorts was performed blindly by a board-certified oral pathologist (Figs. 8c, d; S4d, e). Immunostaining of *Kdm1a*^*−/−*^ + 4NQO mice and SP2509 treated mice tongue sections showed inhibition of phosho-STAT3 (Fig. 9a). To evaluate the CTLA4+ immune cell population, we performed immunostaining with an APC anti-mouse CD152 (CTLA4) antibody on *Kdm1a*^*fl/fl*^ and *Kdm1a*^*−/−*^ mouse tongues. We observed a significant decrease in CTLA4+ immune cells after *Kdm1a* deletion (Figs. 9b; S4f). To further evaluate the effect of *Kdm1a* knockout, we performed a co-culture experiment with CRISPR-Cas9 knockout (*KDM1A*^*−/−*^) in HSC3 cells and observed a significant decrease in CTLA4+ immune cells (Fig. S4g). This finding suggests a functional relationship between LSD1 and CTLA4 in OSCC.

### *Kdm1a* knockout results in a reduced *Stat3* and STAT3-related protein network

**Fig. 10:**
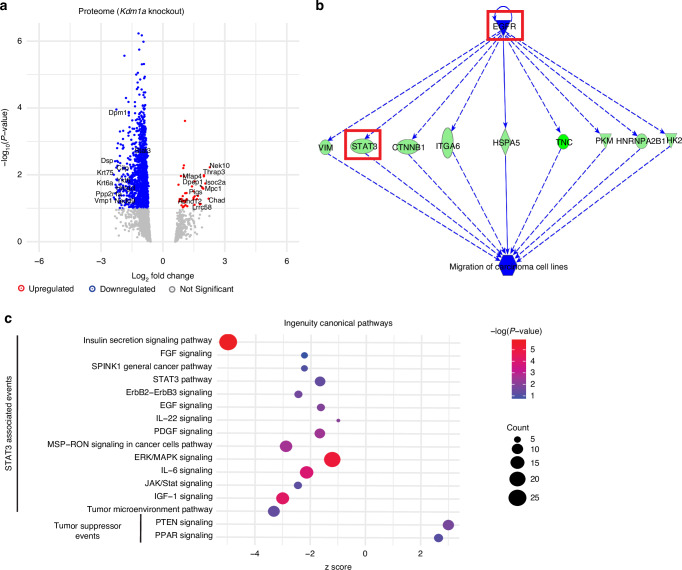
Proteomics analysis showing Kdm1a knockout impairs STAT3 protein network: Global proteomics of tongue protein lysate from *Kdm1a*^*−/−*^ + 4NQO compared to *Kdm1a*^fl/fl^ + 4NQO. **a** Volcano plot showing downregulation of STAT3 along with top dysregulated genes. **b** IPA analysis of candidate genes involved in EGFR-related network. **c** IPA analysis shows affected STAT3-associated events and well as increased tumor suppressor events

To evaluate changes in the overall microenvironment via an unbiased approach, we performed global proteomic analysis of protein lysates isolated from the tongues of *Kdm1a*^*fl/fl*^ and *Kdm1a*^*−/−*^ mice at week 18 post-4NQO treatment (*n* = 7/condition) (Table S6). *Kdm1a* deletion in 4NQO mouse tongue lysate reduced STAT3 protein expression (Fig. 10a). Moreover, differential expression analysis followed by IPA revealed a dysregulated IL6-JAK2-STAT3 network, including the nuclear translocation of STAT3 (Fig. S5a) and EGFR-STAT3 network (Fig. 10b), which are key promoters of OSCC. Furthermore, STAT3 associated events were reduced (Figs. 10c; S5b), thus validating our finding that LSD1 regulates STAT3 and its pathways. Therefore, it can be concluded that LSD1 promotes STAT3 and STAT3-related networks through a novel key oncogenic mechanism.

### SP2509 attenuates total STAT3 network proteins activity

**Fig. 11:**
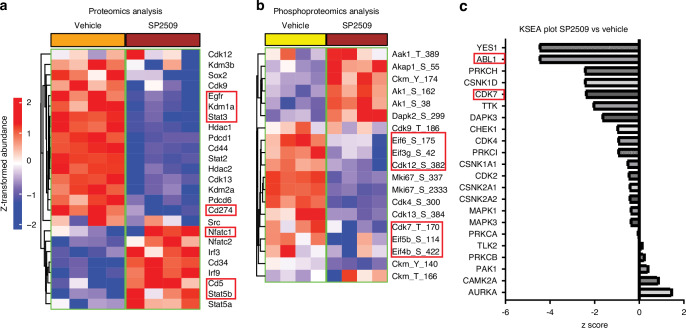
Proteomics analysis showing LSD1 inhibition impairs STAT3 protein and phospho-protein network: **a** Global proteomics of tongue tumor protein lysate from 4MOSC1 syngeneic mouse model showing SP2509 treatment reduces LSD1 and STAT3, whereas increased NFATc1 and IRF3. **b** Phosphoproteomics analysis of 4MOSC1 tumors treated with SP2509 reversed phosphorylated oncoproteins expression shown in the heat map, including phospho-CDK7 (Tyr170). **c** Kinase-substrate enrichment analysis (KSEA) in Phosphomatics tool reveals the kinase activity-based z-score (activation/deactivation) on the reduced activity for phosphorylated CDK7

**Fig. 12:**
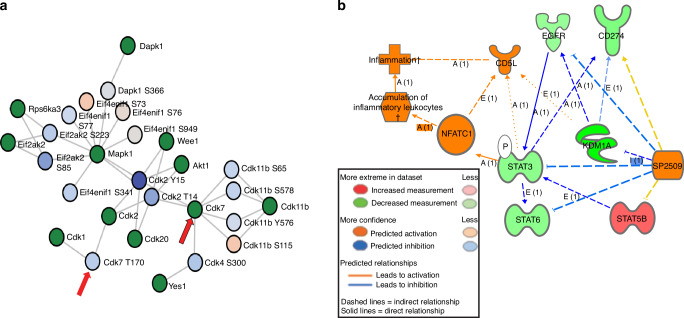
Phosphoproteomics analysis showing LSD1 inhibition impairs CDK7 and EGFR-STAT3 network: **a** Kinase substrate interaction analysis in SP2509 treated groups shows inhibition of CDK7 phosphorylation, which has various substrates, including other CDKs and eukaryotic translation initiation factors. **b** IPA analysis generated by global proteomics data shows that SP2509 reduces the EGFR-STAT3 network, whereas upregulation of NFATc1 results in accumulation of inflammatory leukocyte network

Global proteomics analysis showed that SP2509 attenuates various epigenetic regulators (LSD1, HDAC1, HDAC2, and KDM3B), cyclin-dependent kinases (CDK9, CDK12, and CDK13), immune regulators (PDCD1 and CD274), and STAT3. SP2509 increased the levels of specific immune regulators, such as IRF3, IRF9, CD34, CD5, STAT5A, and STAT5B (Fig. 11a, Table S7). Phosphoproteomic analysis showed that SP2509-treated OSCC cells inhibited various cyclins at specific functional sites, including (CDK12 at Ser382, CDK4 at Ser300, CDK13 at Ser384, CDK7 at Tyr170, CDK9 at Tyr186), proliferation markers (Mki67 at Ser337, and Ser2333) and eukaryotic initiation factors (eIF3G, eIF4B, eIF5B, and eIF6) (Fig. 11b, Table S8). Kinase-substrate analysis (KSEA) showed that LSD1-mediated inhibition of phospho-STAT3 also attenuated cyclin-dependent kinases involved in cell cycle regulation (Fig. 11c). A negative z-score indicates inhibition of kinase activity. Phosphomatic predictive analysis of phosphoproteomics data showed that the CDK2-CDK7 interaction was inhibited, resulting in reduced CDK7 activity in the overall network (Fig. 12a) (Fig. S6a). The Search Tool for the Retrieval of Interacting Genes/Proteins (STRING) (Fig. S6b) and IPA (Fig. 12b) (Fig. S6c) analysis showed that the inhibition of LSD1 promoted an increase in NFATc1, accumulation of inflammatory leukocytes, and an inflammatory response, whereas it inhibited the EGFR, STAT3 network, and CD274 expression.

### LSD1 promotes CDK7, which induces STAT3-related OSCC preneoplasia

**Fig. 13:**
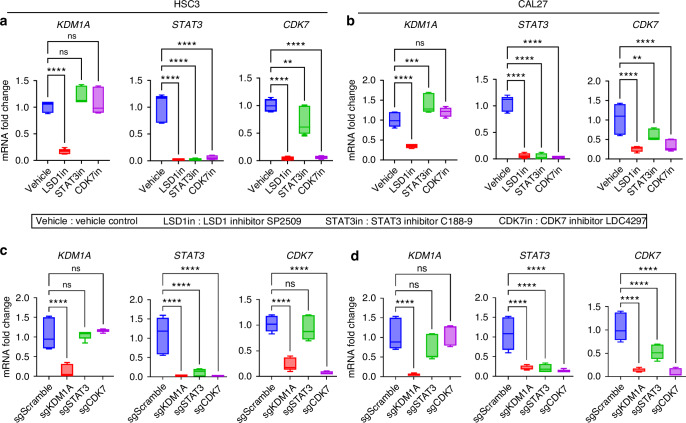
CDK7 is a key mediator of LSD1-induced STAT3 expression: RT-qPCR analysis to evaluate the effect of LSD1, STAT3, or CDK7 inhibitors on expression *KDM1A, STAT3*, and *CDK7* expression in; **a** HSC3 cells, and **b** CAL27 cells. RT-qPCR analysis to evaluate the effect of genetic knockout of *KDM1A, STAT3*, or *CDK7* on expression of *KDM1A, STAT3*, and *CDK7* in; **c** HSC3 cells, and **d** CAL27 cells. “ns” *P* > 0.05, *P < 0.05, **P < 0.01, ***P < 0.001, ****P < 0.000 1

**Fig. 14:**
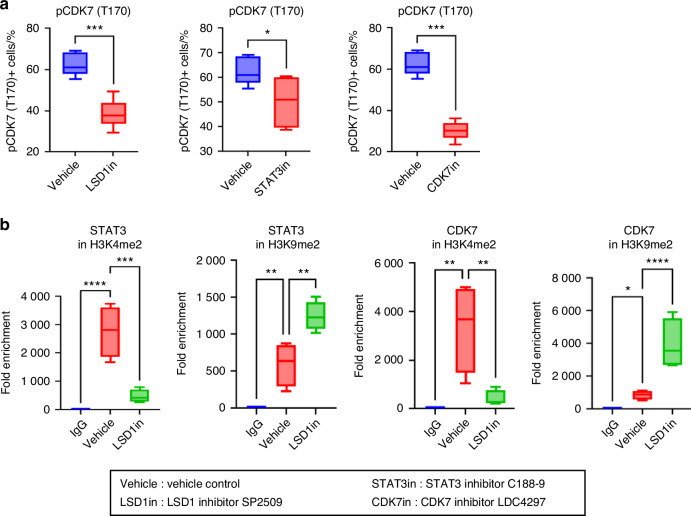
Impact on phosphorylation of CDK7 and methylation of H3K4 and H3K9 after LSD1 inhibition: **a** Effect on phospho-CDK7 (T170) after LSD1, STAT3 and CDK7 inhibition. **b** Status of H3K4 and H3K9 methylation on STAT3 and CDK7. Statistical analysis was performed by *t*-test and one-way ANOVA. “ns” *P* > 0.05, *P < 0.05, **P < 0.01, ***P < 0.001, ****P < 0.000 1

We used specific inhibitors of LSD1, STAT3, and CDK7 to understand the regulatory relationship in OSCC cell lines HSC3 and CAL27 (Fig. 13a, b). LSD1 inhibitors attenuated the expression of KDM1A, STAT3, and CDK7, STAT3 inhibitors inhibited STAT3 only, and CDK7 inhibitors attenuated STAT3 and CDK7. Thus, we identified that LSD1 inhibition attenuates CDK7 activating phosphorylation (Fig. 13b) and CDK7 expression (Fig. 13a, b), leading to STAT3 inhibition, where STAT3 promotes immunosuppression and OSCC preneoplasia progression to OSCC. Similar findings were observed when *KDM1A, STAT3,* and *CDK7* were knocked out using the CRISPR-Cas9 system in HSC3 and CAL27 cells. Briefly, *KDM1A, STAT3,* and *CDK7* were significantly downregulated upon *KDM1A* depletion. However, *STAT3* depletion did not show a synergistic effect on *KDM1A* and *CDK7*, and *CDK7* depletion only affected STAT3 expression (Fig. 13c, d). Next, LSD1 and CDK7 inhibition resulted in significant changes in pCDK7(T170) levels compared to STAT3 inhibition (Fig. 14a). Furthermore, to evaluate the methylation status of STAT3 and CDK7, we performed ChIP analysis and found that the binding of STAT3 and CDK7 with H3K4me2 was significantly reduced after LSD1 inhibition, whereas binding with H3K9me2 was significantly increased (Fig. 14b). Thus, LSD1 affected the chromatin states of STAT3 and CDK7.

## Discussion

Previous studies have shown that LSD1 is a key promoter of OSCC;^12,16^ however, the mechanisms underlying its role in OSCC progression are not fully understood. This study aimed to investigate the role of LSD1 in OSCC progression, and the potential of LSD1 inhibition as a therapeutic strategy. Our unbiased proteomics and transcriptomics approaches identified LSD1 as a key regulator of the CDK7-STAT3-CTLA4 axis in OSCC precancers, which has not been shown in any other study. In addition, we used feline OSCC patients to evaluate the clinical candidate, Seclidemstat (an LSD1 inhibitor), which has translational importance. Additionally, the feline model for evaluating anticancer drugs can be used as a reference for OSCC. *KDM1A* knockout has been demonstrated to reduce both *STAT3* and *CDK7*, whereas *STAT3* knockout has no effect on *KDM1A expression and CDK7* knockout attenuates *STAT3*. This establishes a chain of events in which *KDM1A* regulates *CDK7* and *CDK7* regulates *STAT3* expression. *KDM1A* and *STAT3* were significantly higher in cancer cells than in normal cells. A study showed that SP2509 attenuates STAT3 in DU145 prostate cancer cell lines in vitro and in nude mice.^39^ However, the role of LSD1 in regulating STAT3 mediated by CDK7 is not known in HNSCC and other cancer types. Interestingly, we evaluated the detailed mechanism by which LSD1 inhibition regulates the phosphorylation of CDK7 during HNSCC progression and LSD1 regulates STAT3 and CDK7 by regulating the methylation status of H3K4 and H3K9, which are unique findings demonstrating the specific role of LSD1 in HNSCC.

Previously, a direct interaction has also been observed between LSD1 and STAT3 using affinity capture western blotting.^40^ Our study showed that LSD1 regulates CDK7, STAT3, and CTLA4, key players in cell proliferation and immune suppression, compared to the STAT3 inhibitor alone. Additionally, baseline phosphorylated STAT3 and total STAT3 levels have been proposed as predictive biomarkers for the clinical drug ruxolitinib in patients.^41^ The STAT3 antisense nucleotides, decoy receptors, and STAT3 sh2 domains (NCT02549651, NCT00955812, and NCT00696176, respectively) have been tested in clinical studies.^42^ STAT3 inhibitor and *STAT3* knockout had no significant effect on *KDM1A* expression. Thus, our data suggest that LSD1 inhibition may be a more viable option than STAT3 inhibition alone. However, LSD1 inhibitors need to be compared with STAT3 inhibitors in clinical trials. We demonstrated that LSD1 attenuation inhibited STAT3 at both mRNA and total protein levels. Our earlier studies showed that LSD1 acts as a histone demethylase, promoting demethylation at the H3k4 level,^16^ and by others.^43^

Our study showed that treatment with the LSD1 inhibitor SP2509 reduced cancer cell division. The cell cycle is controlled by cyclin-dependent kinase (CDK) activity, and CDK7 inhibitors have been proven to be effective in cancer treatment.^44^ CDK7 is a key regulator of RNA polymerase II (RNA Pol II)-dependent transcription via the phosphorylation of RNA Pol II and CDK9.^45^ CDK7 is activated upon phosphorylation of T170, which is associated with cell growth,^46^ was reduced upon SP2509 treatment advocating the association between LSD1 and CDK7 phosphorylation. The CDK7 inhibitor YKL-5-124 activates proinflammatory IFNγ signaling and predominantly disrupts cell cycle progression, causing DNA replication stress and genome instability in small-cell lung cancer (SCLC), while simultaneously triggering immune response signaling and provoking T-cell responses.^47^ Interestingly, LSD1 inhibition resulted in elevated IFNγ production in T cells as well as G0/G1 cell cycle arrest, while restricting the transition to the S-phase, which mostly involved DNA replication. Transcription-associated CDK7 promotes initiation and transcription by regulating eukaryotic translation initiation factors (eIFs) such as eIF4B and eIF5B, which promote cancer and immunosuppression.^48,49^ CDK13 directly phosphorylates eIF4B at S422 and promotes tumorigenesis.^50^ Our study showed that SP2509 treatment reduced the activities of eIF4B, eIF5B, and other initiation factors. Overall, our findings indicate that SP2509 attenuates cell division to promote an anticancer phenotype by inhibiting CDK7 activity. Impaired CDK7 activity can inhibit CDK4, CDK9, and CDK13, which act on eIF5B, eIF3G, eIF6, and eIF4B, respectively. Therefore, it can be concluded that LSD1 promotes the phosphorylation of key CDKs and eIFs.

STAT3 plays a role in immune modulation and upregulates the immune checkpoint molecule CTLA4.^21,51^ However, our study introduces a new dimension in which LSD1 inhibition attenuates STAT3-induced signaling and subsequently decreases CD25+ CTLA4+ immunosuppressive cells. These changes may be responsible for CD8+ T-cell infiltration. CD25+ CTLA4+ expression in CD4+ T cells imparts an immunosuppressive phenotype. SP2509 alters the levels of immunosuppressive CD25+ CTLA4+ T cell types and promotes CD4+ and CD8+ T cell infiltration in mouse tongue OSCC. Interestingly, SP2509-treated mice showed upregulation of IFNγ, IFNβ, and IL9 in serum. The spleen also showed an increase in the numbers of CD8+ and CD4+ T cells. Overall, LSD1 inhibition attenuates the immunosuppressive phenotype, which is critical in “cold tumors” such as OSCC, highlighting a specific dual mechanism by which LSD1 inhibition attenuates cancer cell division and promotes the immune response network. IRF3 is required for T cell effector function^52^ to promote IFNγ-induced antitumor immunity to melanoma.^53^ IRF3 also inhibits colorectal^54^ and gastric cancer.^55^ These studies are consistent with our finding that LSD1 inhibition promotes IRF3 expression in OSCC cells.

In addition, we conducted a pilot feline veterinary clinical trial of owned feline spontaneous clinical OSCC patients. We used Seclidemstat (SP2577), an analog of SP2509 that has been extensively studied for its clinical safety (NCT03600649). Our results showed the dual role of Seclidemstat, where it attenuates STAT3 expression and the STAT3-related network and promotes inflammatory leukocyte pathways. The safety of Seclidemstat was also tested in a feline patient with a surgically resected tumor. Seclidemstat did not show any adverse effects. An increased AST/ALT ratio could be a predictor of OSCC, as shown in clinical HNSCC studies.^35–38^ and, interestingly, it was reduced with Seclidemstat treatment. In addition, no visible relapsed or refractory OSCC was detected during the six-month trial period. This study demonstrated that the findings from murine models strongly correlated with those from the feline spontaneous OSCC model. Analysis of publicly available human clinical data showed that networks that are inhibited by LSD1 inhibition are upregulated in patients with OSCC. Our finding that LSD1 promotes the STAT3 network and modulates the infiltration of CD8+ T cells in the tumor microenvironment also correlates with the overall survival of patients with OSCC from TCGA data. Thus, LSD1 inhibition may restore OSCC preneoplasia cells to a relatively normal state.

Our study showed that the LSD1-CDK7-STAT3 network promotes the expression of CTLA4 (CD152) in OSCC preneoplasia. Although these were preclinical studies, they could provide insights into the design of clinical trials involving CTLA4-targeted therapy success and failure. Ongoing clinical trials are targeting CTLA4 in Head and Neck Cancer (NCT04290546 and NCT03690986). The study tested combinations of programmed cell death protein 1 antibody (nivolumab) versus a combination of nivolumab and ipilimumab (NCT02823574) and did not show significant benefits^56^; however, the involvement of the LSD1-CDK7-STAT3 mechanism contributing to resistance is not known. Another study used nivolumab and nivolumab plus ipilimumab in a phase 2 clinical trial of 29 patients randomized trial.^57^ The study concluded that both nivolumab and nivolumab plus ipilimumab were feasible in a neoadjuvant setting and resulted in promising response rates. In addition to regulating CTLA4, LSD1 inhibition also inhibits the expression of PD1 in CD8+ T cells and PD-L1 in epithelial cells. Similar findings were observed in mice treated with the anti-PD1 antibody. Thus, understanding the LSD1 regulated mechanism could help in combination therapies for OSCC preneoplasia in future clinical trials.

## Conclusion

**Fig. 15:**
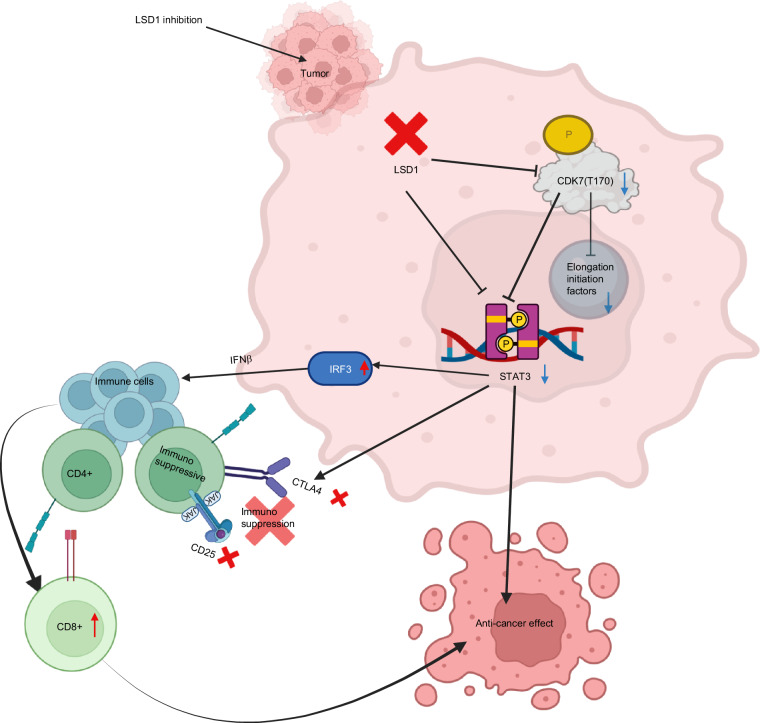
Graphical Abstract. The potential mechanism after blocking LSD1 inhibits novel CDK7 phospho-protein networks and STAT3 signaling ultimately promotes CD8+ T cell infiltration and activation by relieving CTLA4-mediated immunosuppression

Our study sheds light on the crucial role of LSD1 in OSCC progression and provides evidence for the potential use of LSD1 inhibition as a therapeutic strategy for this type of cancer. We showed for the first time that blocking LSD1 inhibits CDK7 phospho-protein networks, leading to the inhibition of STAT3 signaling, which in turn regulates cell cycle progression. Inhibition of the LSD1-CDK7-STAT3 axis promoted CD8+ T cells by relieving CTLA4-mediated immunosuppression (Fig. 15 for graphical abstract). In a pilot veterinary clinical trial, seclidemstat was shown to be safe. Overall, our study demonstrated that LSD1 inhibition has translational applications in OSCC neoplasia. Future research in this field should focus on exploring the molecular mechanisms underlying the role of LSD1 in OSCC and investigating the efficacy of LSD1 inhibition in clinical settings. The findings of this study are limited to animal models and may not fully reflect the complexity of OSCC in humans, which requires further clinical evaluation.

## Materials and methods

### 4NQO mouse model

All experiments were performed with prior approval from the Institutional Animal Care and Use Committee (BUMC IACUC) at Boston University. C57BL/6 J mice were fed 100 μg/mL 4NQO (in propylene glycol) in drinking water for 16 weeks, followed by regular drinking water for the remainder of the study period. Exposure of tongue epithelia to 4NQO results in early and advanced stages of the disease, including hyperplasia (weeks 0–8), papilloma/dysplasia (weeks 9–18), and OSCC (weeks 18–25).^58^ This model captures pathological changes similar to those observed in human OSCC.^26^

### Pilot veterinary trial with naturally occurring feline OSCC

Client-owned cats diagnosed with OSCC and a visible tongue were enrolled in the study trial and received Seclidemstat (SP2577; 10 mg/kg) orally once daily. The first study was conducted to determine the safety and relapse rate of OSCC. A second study was performed to evaluate this effect in the short-term. Tumor biopsy samples were collected before and after treatment and subjected to RNA-seq analysis as previously described. The veterinary trial was conducted in accordance with the NIH Guidelines for the Care and Use of Laboratory Animals, with approval from the University of Florida IACUC Committee (IACUC 202200000137).

### *Kdm1a* knockout mice

LSD1-deficient mice were generated by crossing conditional floxed mice [loxP-Lsd1-loxP^59^] with K14 promoter-driven tamoxifen-inducible Cre mice (K14Cre^ERT^; Jackson Laboratory, stock #005107). The mice were fed 4NQO in drinking water. Tamoxifen was administered to the tongue ten weeks post-4NQO (preneoplasia), and the mice were sacrificed at week 18. K14 promoter-driven conditional Kdm1a-floxed mice treated with vehicle were designated *Kdm1a*^*fl/fl*^, whereas tamoxifen-treated mice were designated as *Kdm1a*^*−/−*^.

### 4NQO-primary tumor cells syngeneic mouse model

4MOSC1 primary cells were extracted by microdissection of 4NQO treated mice 4NQO1 obtained from Gutkind laboratory, and UCSD was used to study OSCC in mice.^26^ Seven-week-old C57BL/6 male and female mice were randomized into three groups (*n* = 10/condition) and implanted with 250 000 4MOSC1 cells, using a previously published protocol.^26^ The mice were treated for three days post-implantation with 1) vehicle (25 µL corn oil, 5% DMSO) or 2) SP2509 (40 mg/kg) five times a week for four weeks. The mouse tongue was measured using calipers at intervals of 4 days throughout the experiment. At the time of sacrifice, tongue tumors were cut into three parts: total RNA, histology, and proteomic analysis.

### Cell culture

HSC3 and CAL27 cells were grown in 6-well plates in DMEM, 10%FBS, and penicillin-streptomycin for 24 h, followed by treatment with vehicle or LSD1 inhibitor (SP2509), and flow cytometry for phospho-STAT3. To evaluate mRNA expression, the cells were treated with vehicle or LSD1 inhibitor (SP2509), STAT3 inhibitor (C188-9)^60^, and CDK7 inhibitor (LDC4297)^61^ purchased from MedCemExpress and added to the respective groups at a final concentration of 1-3 µmol/L final concentration in the respective groups, followed by total RNA extraction and RT-qPCR. For genetic knockout, we used plasmids *CDK7* sgRNA (BRDN0001162216) (Addgene #76077), EF.STAT3C.Ubc.GFP (Addgene #24983), *KDM1A* sgRNA CRISPR/Cas9 All-in-one Lentivector (abm #K2776607).

For the co-culture model, 100 000 HSC3 cells per well were grown in a 6-well plate in DMEM, 10% FBS, and 1% penicillin-streptomycin overnight and treated as follows: 1) vehicle, 2) SP2509 (1 µmol/L), 3) scrambled sgRNA, or 4) sg*KDM1A*/sg*CDK7*/sg*STAT3* for 24 h. For each group. The medium was then replaced with fresh medium, followed by the addition of 20 000 PBMCs and incubation for another 24 h. Finally, the cells were fixed and stained using a flow cytometer.

### RNA extraction and analysis

Total RNA was extracted using TRIzol reagent. RNA-seq and gene set enrichment analyses were performed as described in our previous studies using 400 ng of total RNA for sequencing using Novoseq.^12,16^ Raw FASTQ sequencing reads were mapped against the reference genomes of Mus musculus (mm10), feline (Felis catus; ASM18133v1), and humans (Hg38). Differential gene expression analysis was performed using DESeq2 in the R/Bioconductor software. Hub genes were identified using the CytoHubba plugin in Cytoscape.^62^

### Pathological characterization and immunostaining

Tongue sections were stained with H&E and evaluated for pathology by a board-certified pathologist. Immunostaining and H&E staining were performed on *n* = 5-8/group and four sections/followed, which induced OSCC that mirrors the progressive onset of the human disease. Mouse tissue sections were stained with anti-LSD1 antibody (Abcam: ab17221) or anti-phospho-STAT3 (Tyr705; Abcam) antibodies in the respective groups, and images were quantified using the ImageJ software (NIH).

### Public data analysis

CPTAC data were accessed using the University of Alabama at the Birmingham CANcer (UALCAN) Portal, and TCGA data were obtained using the TCGABiolinks R/Bioconductor package. Differential expression analysis for TCGA and precancer data was performed using edgeR and limma pipelines, respectively. Kaplan-Meier survival estimation was performed using cBioPortal. Only OSCC samples were considered for survival analysis. First, we filtered the samples using p16 HPV testing, followed by filtering using the HPV test. Approximately 40 samples were HPV-ve, one was HPV+ve, and the remaining 273 samples did not show HPV status; hence, they were considered for this analysis along with HPV-ve. The analysis was performed based on the z-score of the total mRNA expression levels. The total number of samples collected from each oral region is listed in Table S2.

### Proteomics analysis

The protein concentration in each sample was determined using the BCA Protein Assay Kit (Pierce). Proteins (200 μg) were processed, precipitated, digested with 2.5 μg trypsin per sample for 16-hour incubation at 37 °C, and desalted using C18 spin columns (Pierce). The TMT-16 plex kit was used for TMT-based quantitative analysis. One hundred micrograms from the pooled mixture of each set were earmarked, while the remainder was allocated for phosphopeptide enrichment. Global and phosphoproteomic analyses were conducted using Partek Genomics Suite 7, Phosphomatic for kinase-substrate analysis, and Phosphosite Plus to identify the functional phosphorylation sites. Additionally, Ingenuity Pathway Analysis (IPA) was used to assess enrichment networks and signaling pathways.

### Flow cytometry and immunostaining

In this study, we employed five laser 64-color Cytek Aurora spectral flow cytometers to analyze the immune cells isolated from mouse tongue tissues. The tissue was digested with Collagenase P (Sigma, #11249002001) to prepare a single-cell suspension. The cells were washed once with cold PBS at 1 200 r/min for 10 min at 4 °C. The cells were stained with Zombi UV (BioLegend #423108) for 10 min at 4 °C followed by washing with cold FACS buffer. For surface staining, cells were stained with fluorochrome-conjugated antibodies obtained from BioLegend, including BV650 anti-mouse CD45 (#103151), BV421 anti-mouse CD3ε (#100335), PE Cy7 anti-mouse TCRβ (#109221), BV785 anti-mouse CD4 (#100453), PE Dazzle594 anti-mouse CD8 (#100761), PerCP/Cy5.5 anti-mouse CD152 (CTLA4) (#106316), APC anti-mouse CD25 (#101909), PE/Fire™ 700 anti-mouse CD279 (PD-1) antibody (#135268), and Brilliant Violet 605 anti-mouse CD274 (B7-H1, PD-L1) antibody (#124321). For internal staining with PerCP/Cyanine5.5 anti-mouse IFN-γ Antibody (BioLegend #505822), the cells were fixed and permeabilized using a BD Bioscience fixation/permeabilization kit (#554714) according to the manufacturer’s instructions.

For flow cytometry analysis of human OSCC cells and human PBMCs, we used the same BD Bioscience fixation/permeabilization kit (#554714) for fixation and permeabilization, as per the manufacturer’s instructions, and employed Brilliant Violet 605™ anti-human IFN-γ Antibody (#506542), BV510 anti-human CD4 (#300546), FITC anti-human CD8A (#301006), and Alexa Fluor® 647 anti-human CD152 (CTLA-4) antibody (#369626) for surface and internal staining. We used APC anti-mouse CD152 Antibody (#106309) for immunofluorescence staining of the tongues of *Kdm1a*^*−/−*^ mice. Data analysis was performed using FlowJo and the OMIQ software. To analyze STAT3 protein levels in cells by flow cytometry, we used the True-Nuclear™ Transcription Factor Buffer Set (#424401), BioLegend, to fix and permeabilize the cells according to the manufacturer’s protocol and then stained with APC anti-STAT3 antibody (#678014).

### Cell cycle analysis

We seeded 100 000 HSC3 and CAL27 cells per well in 6 well plates and treated them with sgscrambled, sgKDM1A, sgSTAT3, and sgCDK7 48 h after seeding the cells. After 24 h, the cells were fixed with 4% paraformaldehyde, stained with FxCycleTM PI/RNase Staining Solution (Invitrogen #F10797) according to the manufacturer’s protocol, and analyzed using a Cytek Aurora flow cytometer.

### Chromatin immunoprecipitation (ChIP)

ChIP analysis was performed using ~4 × 10^6^ cells and the SimpleChIP® Enzymatic Chromatin IP Kit (Magnetic Beads) according to the manufacturer’s instructions (Cell Signaling Technology, #9003 s). Anti H3K4me2 Recombinant Rabbit Monoclonal Antibody (24H8L19) (Thermoscientific, #701764) and anti H3K9me2 Polyclonal Antibody (Thermoscientific, #39239) were used for ChIP.

## Supplementary information

SuppFig1

SuppFig2

SuppFig3

SuppFig4

SuppFig5

SuppFig6

Supplementary Tables

Supplementary figures

## Data Availability

The authors declare that data supporting the findings of this study are available within the paper and its supplementary information files. Raw RNA-Seq data files are publicly available at NCBI GEO database under the accession IDs GSE277930 (Feline) and GSE277935 (Mice).
