# Supplementary figures and images for: Lysine-specific demethylase 1 controls key OSCC preneoplasia inducer STAT3 through CDK7 phosphorylation during oncogenic progression and immunosuppression
Source: Int J Oral Sci. 2025 Apr 17;17:31. doi: 10.1038/s41368-025-00363-x (PMC12006301; doi:10.1038/s41368-025-00363-x)

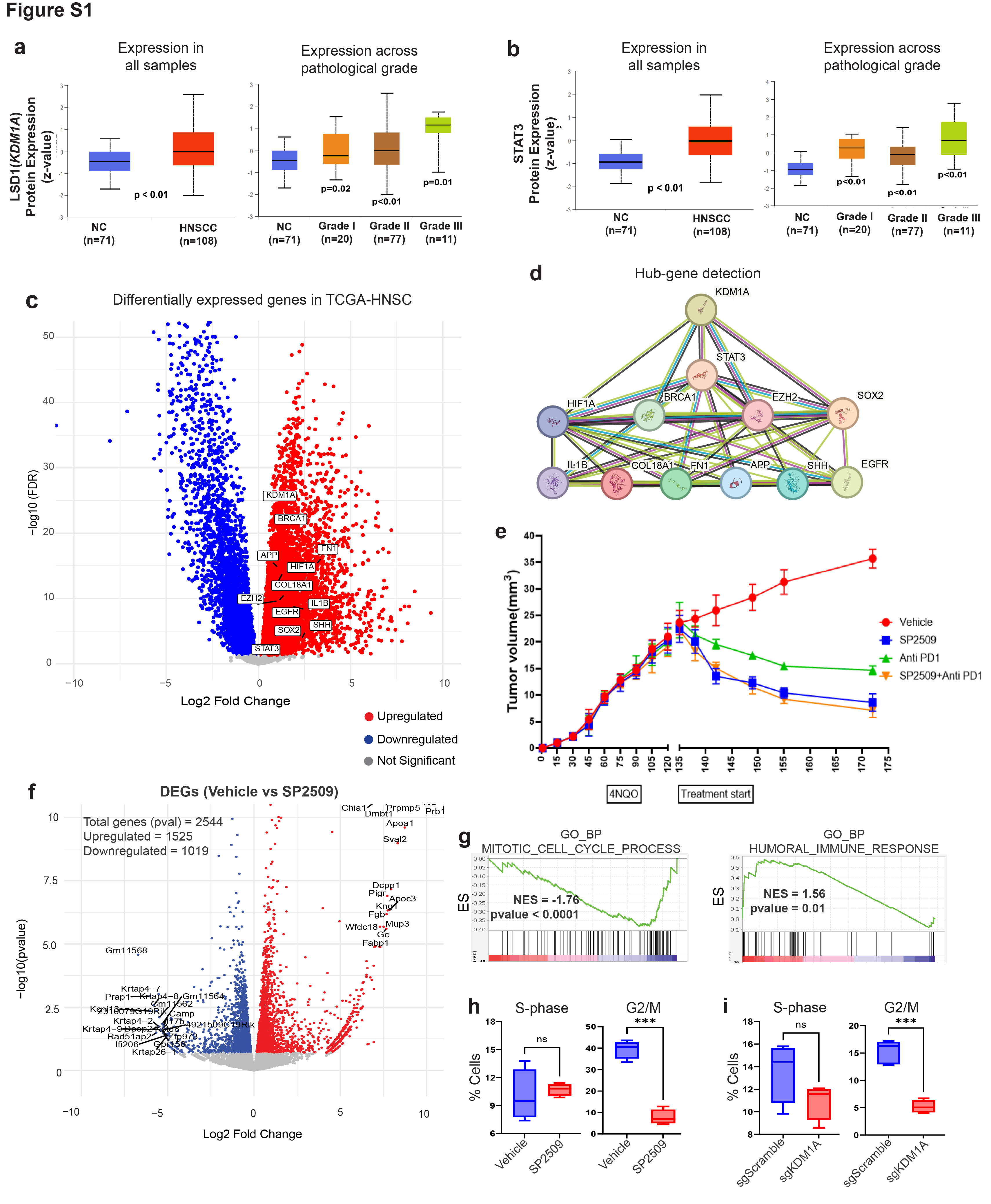

Supplement: Supplementary file 1 — SuppFig1 [file 41368_2025_363_MOESM1_ESM.png]

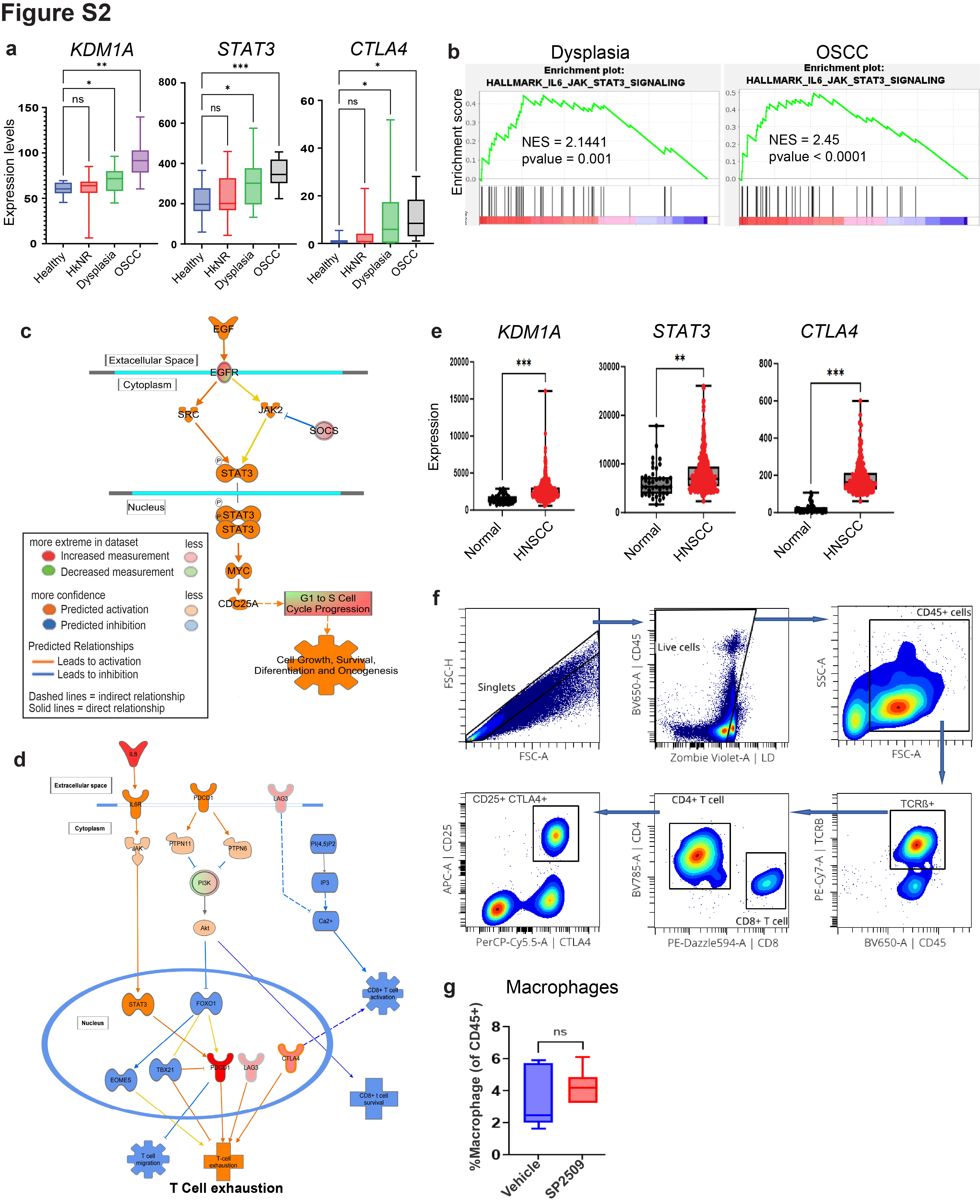

Supplement: Supplementary file 2 — SuppFig2 [file 41368_2025_363_MOESM2_ESM.png]

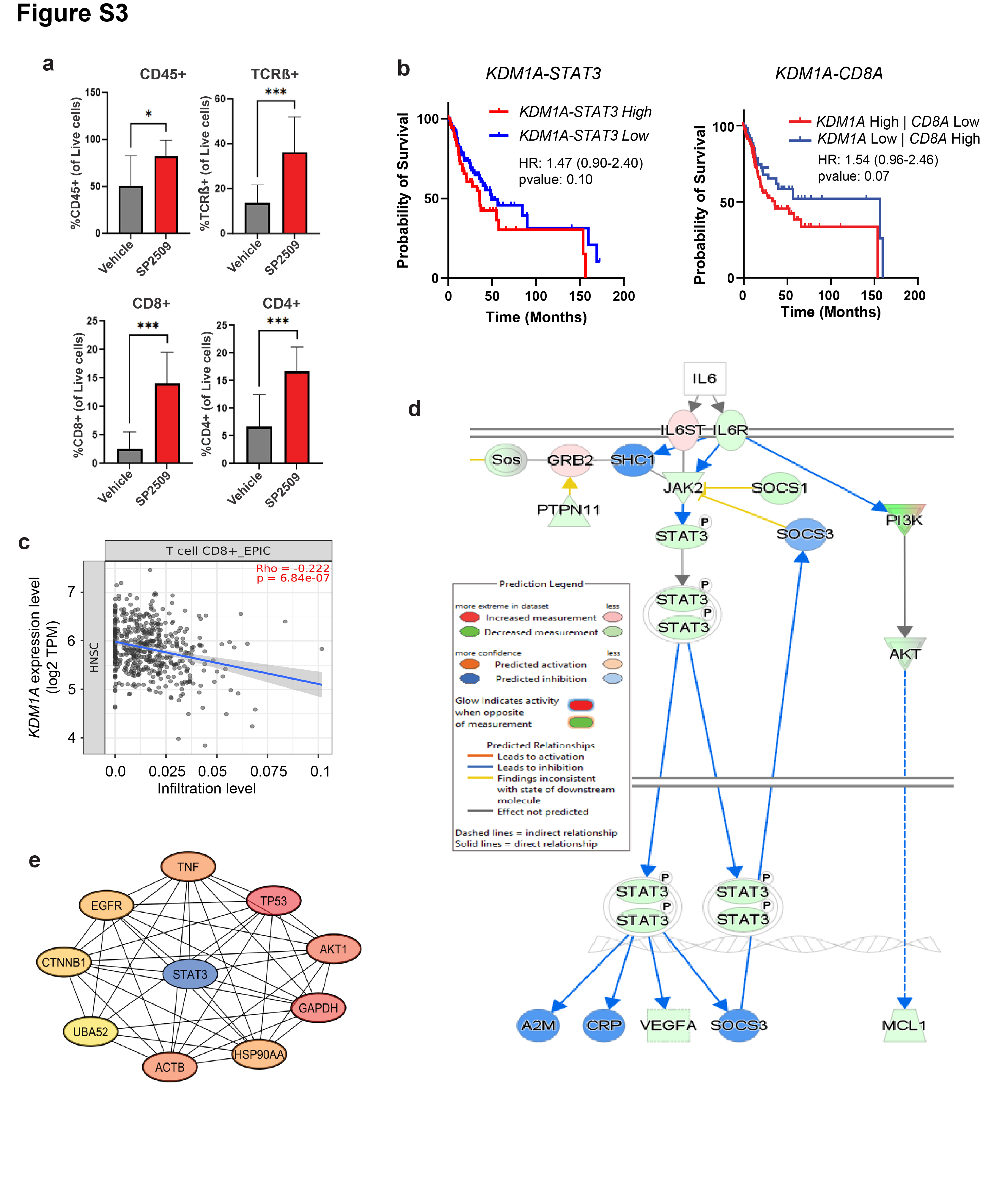

Supplement: Supplementary file 3 — SuppFig3 [file 41368_2025_363_MOESM3_ESM.png]

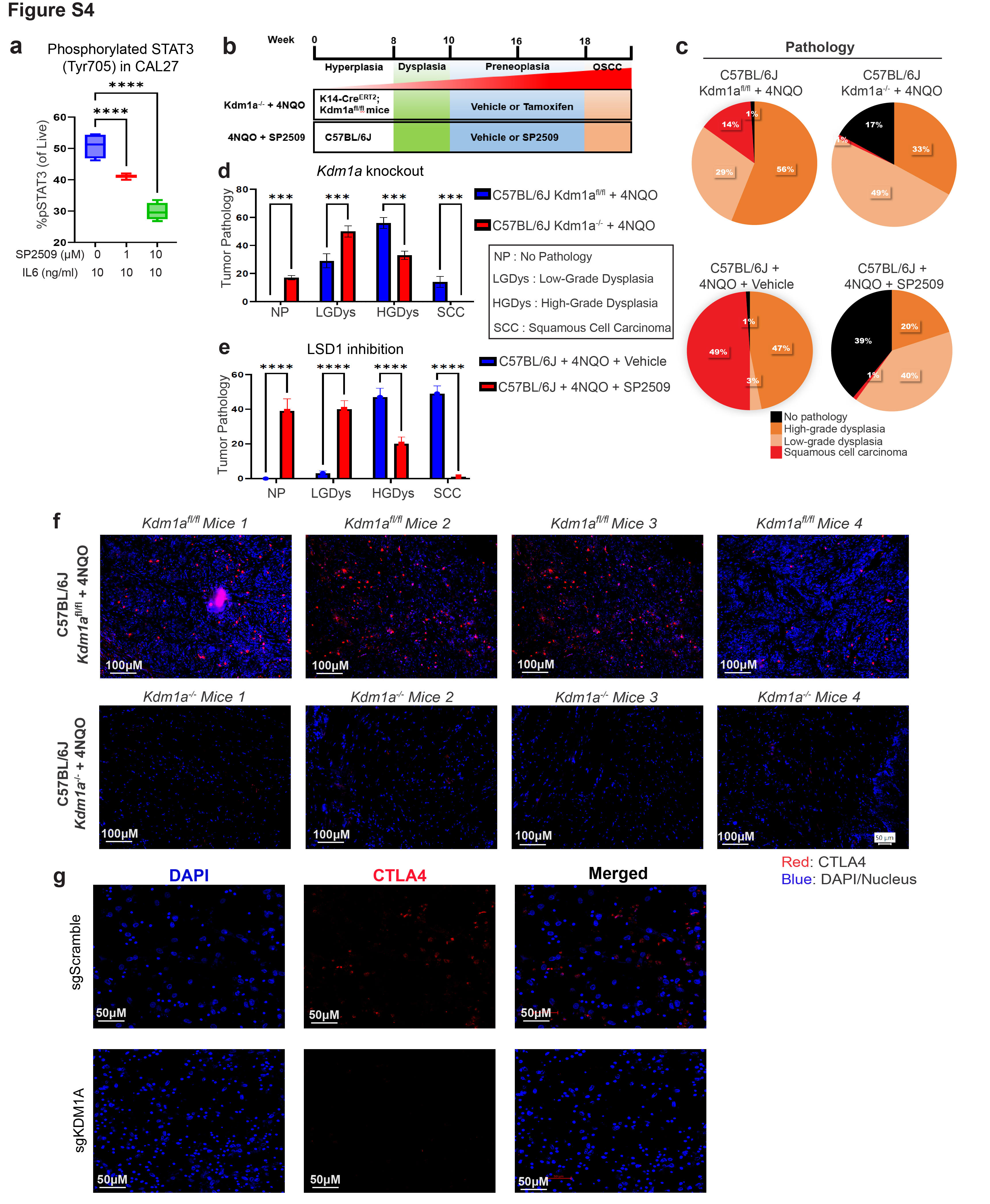

Supplement: Supplementary file 4 — SuppFig4 [file 41368_2025_363_MOESM4_ESM.png]

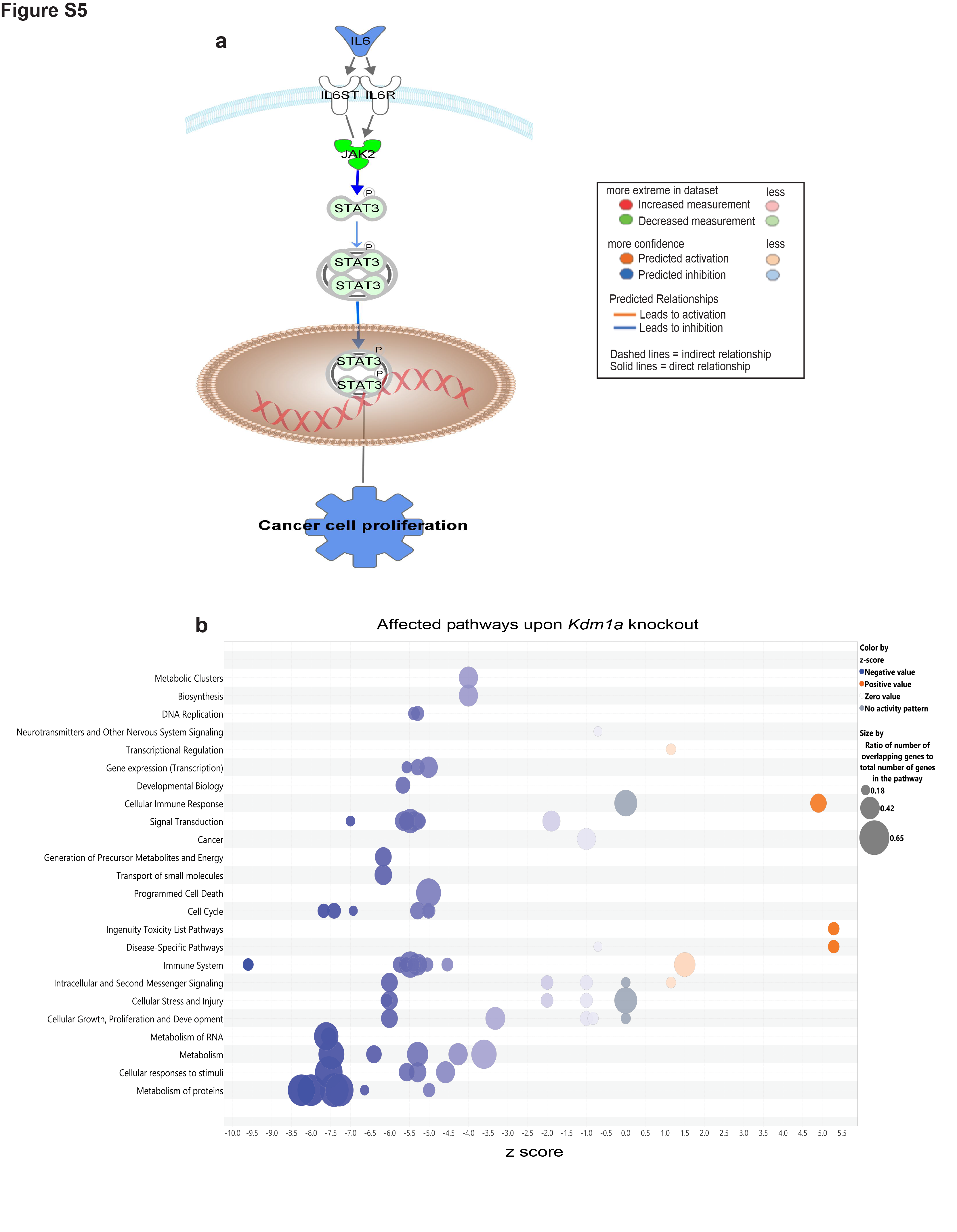

Supplement: Supplementary file 5 — SuppFig5 [file 41368_2025_363_MOESM5_ESM.png]

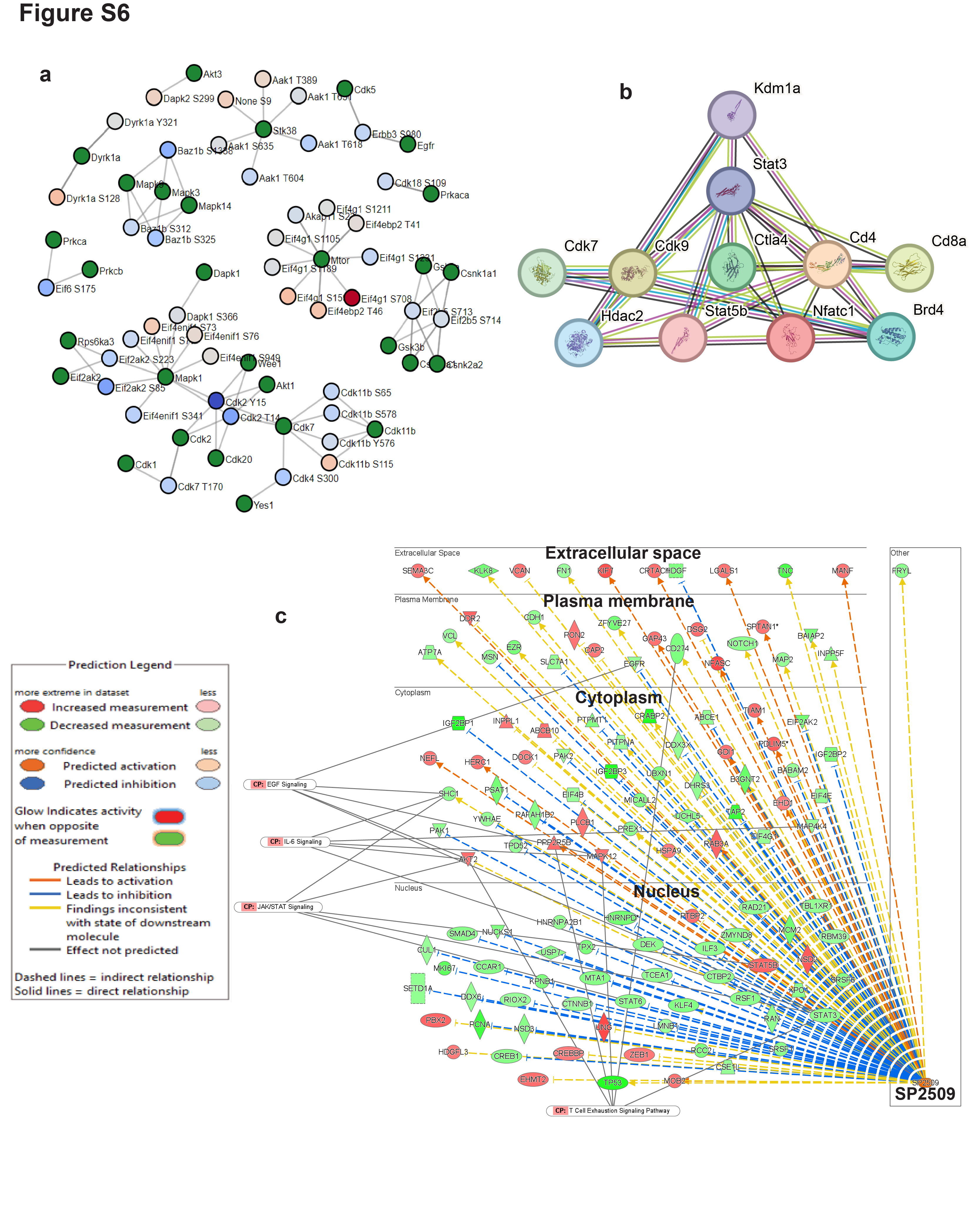

Supplement: Supplementary file 6 — SuppFig6 [file 41368_2025_363_MOESM6_ESM.png]
