# Supplementary material for: Lysine-specific demethylase 1 controls key OSCC preneoplasia inducer STAT3 through CDK7 phosphorylation during oncogenic progression and immunosuppression
Source: Int J Oral Sci. 2025 Apr 17;17:31. doi: 10.1038/s41368-025-00363-x (PMC12006301; doi:10.1038/s41368-025-00363-x)
Supplement: Supplementary file 8 — Supplementary figures [file 41368_2025_363_MOESM8_ESM.docx]

**Supplementary figures:**

**Fig. S1:** a) LSD1 protein expression in HNSCC patients increasing with the tumor grade. b) STAT3 protein expression in HNSCC patients increasing with the tumor grade. c) Differentially expressed genes in TCGA-HNSC data show significantly up- and downregulated gene cohort, including *STAT3* and *KDM1A*. d) Hub gene network analysis using differentially expressed genes from TCGA-HNSC data shows the relationship of *KDM1A* and *STAT3* with top 10 hub genes. e) Tumor growth curve. f) Volcano plot showing SP2509 inhibited and activated genes. G) GSEA plots showing gene enrichments after LSD1 inhibition. Cell cycle analysis showing significant changes observed in G2/M phage of cell cycle in HSC3 cells after treatment with h) SP2509, and i) sg*KDM1A* mediated knockout. “ns” P-value>0.05, * P-value<0.05, ** P-value<0.01, *** P-value<0.001, **** P- value<0.0001.

**Fig. S2:** a) Publicly available precancer patient dataset shows significant upregulation of *KDM1A, STAT3* and *CTLA4* during the progression to OSCC. b) Hallmark analysis shows significant positive enrichment of IL6-JAK-STAT3 pathway related genes in oral dysplasia OSCC patients. c) IPA analysis confirms the highly active JAK-STAT3 pathway leading to activated *MYC* and G1 to S phage cell cycle progression. d) IPA analysis also shows activation of *PDCD1* and *CTLA4* due to activation of *STAT3* network in OSCC leads to exhaustion of T cell. e) Significant upregulation of *KDM1A*, *STAT3* and *CTLA4* in publicly available HNSCC dataset. f) Gating strategy for flow cytometry data to visualize the immune cells in the SP2509 treated mice tongue with OSCC. g) SP2509 treatment did not affect overall macrophage levels in the SP2509 treated mice tongue with OSCC. “ns” P-value>0.05, * P-value<0.05, ** P-value<0.01, *** P-value<0.001, **** P- value<0.0001.

**Fig. S3: a**) Spleen cell analysis with flow cytometry shows significant upregulation of immune cells (CD45+, TCRβ+, CD8+ and CD4+ T cells) in the spleen after treatment with SP2509. b) Survival analysis HPV- OSCC patients from TCGA shows slightly significant increase in overall survival for *KDM1A (low expression)* with *STAT3 (low expression)* as well as *KDM1A (low expression)* with *CD8A (high expression)* conditions in OSCC. c) TIMER 2.0 immune deconvolution analysis of TCGA data shows inverse correlation between *KDM1A* expression and CD8+ T cell infiltration. d) IPA analysis of mouse RNA-seq data shows inhibited JAK-STAT3 network after SP2509 treatment. e) Network showing top 10 hub genes, including STAT3, in Seclidemstat treated feline OSCC patients. “ns” P-value>0.05, * P-value<0.05, ** P-value<0.01, *** P-value<0.001, **** P- value<0.0001.

**Fig. S4:** a) Phosphorylated STAT3 (Tyr705) has been decreased significantly after SP2509 treatment. b) Pictorial representation of the experimental design of the genetic deletion (K14-CreERT2; *Kdm1a*^fl/fl^ and pharmacological inhibition (SP2509) of LSD1 in C57BL/6J mice. C) Pi chart showing overall status of pathological changes in 4NQO induced mice OSCC and treatment. d-e) Quantification showing the significant reduction in dysplasia grades after d) Homozygous *Kdm1a* knockout, and e) SP2509 treatment, in 4NQO induced mice OSCC. f) Immunofluorescence shows significant reduction of CTLA4+ cells in *Kdm1a^-/-^* mice tongue (Red color indicates CTLA4+ immune cells and blue indicates DAPI). g) Immunostaining shows significant reduction in CTLA4+ T cells in *sgKDM1A* treated HSC3 cells co-cultured with human PBMC (Red color indicates CTLA4+ cells and blue color indicates the nucleus). “ns” P-value>0.05, * P-value<0.05, ** P-value<0.01, *** P-value<0.001, **** P- value<0.0001.

**Fig. S5: a**) IPA analysis showing inhibition of JAK2-STAT3 pathway. b) Affected canonical pathways after *KDM1A* knockout in 4NQO induced HNSCC in mice.

**Fig. S6: a**) Extended kinase-substrate interaction network shows effect of LSD1 inhibition in phosphoproteomic data analysis of 4MOSC1 injected mice tumor. b) STRING analysis shows interaction of KDM1A-STAT3 and the downstream genes that have direct relation with KDM1A and STAT3. The experimentally proven relations are only taken into consideration to generate the network. c) IPA generated network from proteomics data showing the affected proteins after SP2509 treatment and their positions in the cell.
